# Prolonged Seizure Activity Followed by Severe Hyperphosphatemia and Hypocalcemia in a Pediatric Patient

**DOI:** 10.7759/cureus.14338

**Published:** 2021-04-07

**Authors:** Megan B Coriell, Andrew T Van Hersh, Siddharth Shah

**Affiliations:** 1 Pediatric Endocrinology, University of Louisville School of Medicine, Louisville, USA; 2 Pediatrics, University of Louisville School of Medicine, Louisville, USA; 3 Pediatric Nephrology, University of Louisville School of Medicine, Louisville, USA

**Keywords:** hypocalcemia, hyperphosphatemia, cell lysis, seizure, rhabdomyolysis, prolonged seizure, electrolyte abnormality, seizure activity, pediatric

## Abstract

Seizures secondary to hypocalcemia have been well documented and studied. There are various causes of hypocalcemia described in the literature, but a prolonged seizure episode leading to cell lysis, severe hyperphosphatemia, and hypocalcemia is rarely reported. We present the case of a 3-year-old male with severe hyperphosphatemia and hypocalcemia secondary to the likely presence of cell lysis from prolonged seizure activity. Our case illustrates the importance of a thorough evaluation of the possible differentials of hypocalcemia and hyperphosphatemia in a challenging presentation.

## Introduction

Seizures in the setting of hypocalcemia have been reported in the literature [1-4]. Different causes of hypocalcemia have been described, including hypoparathyroidism, pseudohypoparathyroidism, and phosphate-containing enema administration. There have also been reports of rhabdomyolysis occurring secondary to seizure activity [5,6]. However, we could not find relevant information in the pediatric literature about cell lysis from prolonged seizure activity leading to severe hyperphosphatemia and hypocalcemia. In this case report, we present a 3-year-old male who presented with severe hyperphosphatemia and hypocalcemia in the setting of prolonged seizure activity.

## Case presentation

Our patient is a 3-year-old male with a significant history of 27-week prematurity, mild cerebral palsy, epilepsy, and chronic constipation. He presented with severe electrolyte derangements in the setting of prolonged seizure activity. The patient had seizure activity that lasted 20-40 minutes on the day of presentation to our hospital. This was the longest seizure he had ever experienced. He had used a homemade saline enema on the day before this event. He was found to have abnormal electrolytes with a serum potassium of 6.8 mmol/L (Ref: 3.5-5.1 mmol/L), serum bicarbonate of 9 mmol/L (Ref: 23-28 mmol/L), serum calcium of 4 mg/dL (Ref: 8.6-11.2 mg/dL), serum phosphorus of >13 mg/dL (Ref: 2.5-4.5 mg/dL), and serum magnesium of 1.2 mg/dL (Ref: 1.6-2.3 mg/dL) at presentation. A head CT was performed, and it was normal. Blood and urine cultures were also obtained. The initial workup was also notable for troponin of 0.1 ng/mL (Ref: 0.000-0.034 ng/mL), ionized calcium of 0.4 mmol/L (Ref: 1.14-1.29 mmol/L), c-reactive protein of 1.4 mg/dL (Ref: <1.0 mg/dL), lactic acid of 3.6 mmol/L (Ref: 0.7-2.0 mmol/L), and procalcitonin of 57.3 ng/mL (Ref: 0.0-0.5 ng/mL).

Diagnostic assessment

Multiple other labs were obtained, including thyroid-stimulating hormone (TSH), free T4, parathyroid hormone (PTH), vitamin D, uric acid, lactate dehydrogenase (LDH), and creatine kinase (CK). Of these, PTH was elevated to 280.7 pg/mL (Ref: 11.5-82.7 pg/mL), LDH was elevated to 1413 U/L (Ref: 313-618 U/L), and CK was elevated to 703 U/L (Ref: 30-170 U/L). TSH was low at 0.109 iU/mL (Ref: 0.470-4.680 iU/mL) with a normal free T4 of 1.14 ng/dL (Ref: 0.78-2.19 ng/dL). This was thought to be due to sick euthyroid given the patient’s acute illness. The 1,25-vitamin D was normal at 55.6 pg/mL (Ref: 19.9-79.3 pg/mL), as was his 25 vitamin D at 26.7 ng/mL (Ref: 20.0-100.0 ng/mL) and alkaline phosphatase at 138 U/L (Ref: 115-460 U/L). There was no evidence of hypoparathyroidism or vitamin D toxicity based on these studies. We suspected that the initial PTH elevation might be secondary to severe hypocalcemia.

Further workup included an adrenocorticotropic hormone (ACTH) stimulation test and urine calcium and phosphorous. ACTH stimulation test results were normal, with 30-minute cortisol of 48.8 ug/dL and 60-minute cortisol of 59.5 ug/dL (Ref: 4.46-22.7 ug-dL), making adrenal insufficiency less likely. Baseline cortisol was not obtained.

Urine calcium and phosphorous excretion were appropriate in response to electrolyte anomalies with low urine calcium (<1.0 mg/dL) and high urine phosphorous (>286.0 mg/dL). The repeat phosphorus after a few hours was further reported at 15.8 mg/dL. Our patient had significant hypocalcemia, hyperphosphatemia, and expected elevation in PTH in the setting of his significant hypocalcemia, along with evidence of cell lysis with high CK and LDH levels following the seizure episode.

Treatment and clinical course

**Figure 1:**
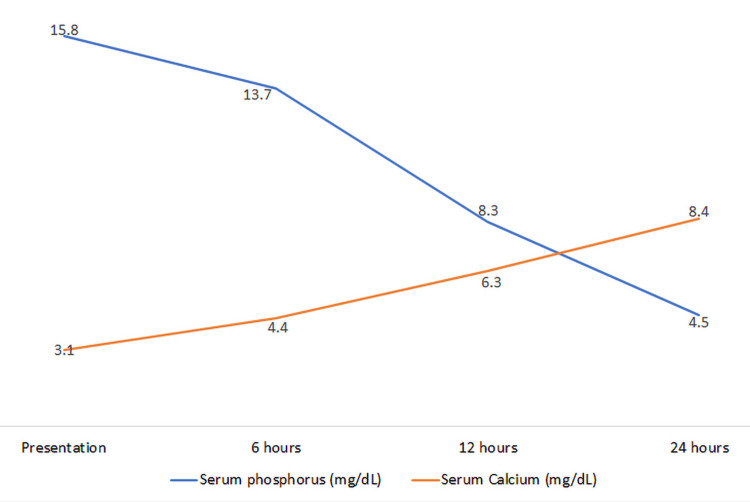
Serum Phosphorous and Calcium Trends During the First 24 Hours of Admission

The patient was admitted to the Pediatric Intensive Care Unit (PICU) for the management of his electrolyte abnormalities with endocrinology and nephrology services actively involved in his care. He received intravenous fluids to help correct his electrolyte abnormalities and acidosis. The calcium was corrected with intravenous calcium gluconate over the course of 24 hours, after which he was transitioned to oral calcium carbonate. He also received intravenous magnesium supplementation for the correction of hypomagnesemia. Sevelamer at the dose of 400 mg PO TID was added to help correct his severe hyperphosphatemia. Ergocalciferol was started for vitamin D supplementation and to help maintain stable calcium levels. The serum calcium and phosphorus improved within 24 hours of admission to the PICU (Figure 1).

Procalcitonin was initially elevated, but it improved over the course of a few days. Of note, elevated procalcitonin has been previously reported in the setting of convulsions [7]. Similarly, the elevated lactate levels returned to the normal range following intravenous hydration. We did not see the presence of persistently high lactic acidosis or evidence of diabetic ketoacidosis that could have contributed to an acute extracellular phosphorus shift.

The family used homemade saline enemas to treat his constipation and there was no recent history of the use of phosphorus-containing enemas. The peak serum creatinine was 0.5 mg/dL and the patient continued to have good urine output during admission. There was no evidence of a significant acute kidney injury episode that could have contributed to phosphorus retention. Also, there was no substantial evidence of the presence of acute phosphate nephropathy. The kidneys had normal size and echogenicity on renal ultrasound. The blood and urine cultures obtained on admission were ultimately negative for infection.

He was able to be weaned off his electrolyte supplementation slowly. The PTH level rechecked on day two of admission had returned to normal at 24.4 pg/mL. The calcium carbonate was weaned off over the course of six days. The patient continued to have normal electrolytes throughout the remainder of his admission and was discharged only on ergocalciferol supplementation.

Follow up

He had no further seizure activity after discharge and calcium levels remained stable on follow up labs. At his endocrinology follow-up appointment one month after discharge, he remained off all calcium supplementation and was only taking vitamin D daily. At that time, his calcium and phosphorus levels remained within normal range, with a normal PTH level. Alkaline phosphatase and vitamin D levels were normal as well.

## Discussion

Our patient presented with seizures along with significant hypocalcemia, hyperphosphatemia, and an elevated PTH. Our leading diagnosis was initially pseudohypoparathyroidism - type 1B (PHP type 1B) given that his PTH was elevated in the setting of hypocalcemia and hyperphosphatemia. There have been case reports of seizures as the initial presentation for a patient with both hypoparathyroidism and pseudohypoparathyroidism [3,8]. Our patient did not have any physical exam findings typically seen with pseudohypoparathyroidism (i.e., he did not have Albright hereditary osteodystrophy (AHO) phenotype - no short stature, no short metacarpals, no round facies). However, pseudohypoparathyroidism type 1B can present with isolated pseudohypoparathyroidism, no AHO phenotype, with or without hypothyroidism presenting later in life. Genetic testing may be negative in some cases, but positive identification of a mutation in GNAS (guanine nucleotide binding protein, alpha stimulating) or other PHP-related genes may help establish the diagnosis [9]. In our patient's case, further workup revealed low urine calcium and high urine phosphorous, which pointed away from this diagnosis. His repeat PTH two days after the initial presentation was normal. We would not expect his PTH to normalize so quickly if he truly had pseudohypoparathyroidism. His 1,25-vitamin D level was normal, which also does not support the diagnosis of pseudohypoparathyroidism.

Our differential also included hyperphosphatemia secondary to receiving multiple sodium phosphate (fleet) enemas for his chronic constipation [10,11]. The excess phosphorous binds the calcium and results in hypocalcemia [12]. In a case report by Marraffa et al., a 4-year-old female had a seizure in the setting of hypocalcemia and hyperphosphatemia after receiving two pediatric fleet enemas [13]. Our patient's seizure episode did occur after enema administration. However, mom reported that they make their own enemas at home with salt and water. They did this due to concerns he would receive too much phosphorous with commercial enemas given the frequency at which he required them. Our patient’s mother reports using fleet enemas infrequently with no history of recent use, making the fleets enema unlikely to be the cause of significant hyperphosphatemia seen at presentation.

There are few cell lysis and rhabdomyolysis reports after prolonged seizure episodes in the literature [6]. However, hyperphosphatemia and hypocalcemia have been reported in the setting of rhabdomyolysis [14]. Our patient was found to have elevated LDH and CK levels along with hyperphosphatemia and hypocalcemia concerning for the presence of cell lysis and rhabdomyolysis. This led to the concern that cell lysis from his prolonged seizure could have resulted in hyperphosphatemia, which then caused the hypocalcemia and appropriately elevated PTH in response to the same. In such cases, the high phosphorus load released from the cells combines with the calcium, resulting in hypocalcemia [15]. The CK levels were not extremely high in our patient’s case because they were checked 18 hours after presentation. We suspect that CK levels would have been significantly higher immediately after the seizure activity. There was no evidence of acute kidney injury, which may have helped with the rapid correction of electrolytes. The other differential for cell lysis apart from seizure-induced rhabdomyolysis could be multi-system inflammatory syndrome secondary to viral pathology. His infectious workup, including a COVID-19 test, was negative.

Our patient had high urine phosphorus and low urine calcium suggesting appropriate phosphorus excretion from the kidneys in the setting of hyperphosphatemia and hypocalcemia. The PTH was appropriately elevated for the given degree of hypocalcemia. Given these findings, there was no concern about retaining phosphorus from the kidneys, and elevated phosphorus was likely primarily related to cell lysis.

Hyperphosphatemia is traditionally managed by restricting dietary phosphorous intake. However, this can be difficult in children as it may limit protein and caloric intake that is needed for proper growth and development. In cases of severe hyperphosphatemia, as seen in our patient, dietary changes alone are not sufficient to lower the phosphorous level quickly. Intravenous hydration is often needed for the correction of acute hyperphosphatemia. Phosphate binders may be used in these cases as well, which usually occur in patients with chronic kidney disease on dialysis. Sevelamer is a non-calcium-based phosphate binder and was used in our patient’s case due to his significant hyperphosphatemia. Sevelamer and other phosphate binders reduce the intestinal absorption of phosphorous, thus helping to decrease serum phosphorous levels [16]. If hypocalcemia is also present, the serum calcium often improves as the hyperphosphatemia is corrected, which we did observe in our patient’s case.

This report's unique findings were the presence of significant hyperphosphatemia (phosphorus at 15.8 mg/dL) following a prolonged seizure report. This level of phosphorus elevation has not been previously reported in a pediatric patient following a seizure episode.

## Conclusions

Our case illustrates the importance of developing a broad differential for causes of hypocalcemia and hyperphosphatemia. Our final diagnosis was ultimately one of exclusion, requiring various other diagnoses to be thoroughly evaluated and ruled out. Prolonged seizures may lead to cell lysis, and electrolyte abnormalities such as hyperphosphatemia and hypocalcemia should be carefully monitored and treated following such an episode.
